# Bayesian Markov Random Field Analysis for Protein Function Prediction Based on Network Data

**DOI:** 10.1371/journal.pone.0009293

**Published:** 2010-02-24

**Authors:** Yiannis A. I. Kourmpetis, Aalt D. J. van Dijk, Marco C. A. M. Bink, Roeland C. H. J. van Ham, Cajo J. F. ter Braak

**Affiliations:** 1 Biometris, Wageningen University and Research Centre, Wageningen, The Netherlands; 2 Applied Bioinformatics, Plant Research International, Wageningen, The Netherlands; 3 Laboratory of Bioinformatics, Wageningen University, Wageningen, The Netherlands; Miami University, United States of America

## Abstract

Inference of protein functions is one of the most important aims of modern biology. To fully exploit the large volumes of genomic data typically produced in modern-day genomic experiments, automated computational methods for protein function prediction are urgently needed. Established methods use sequence or structure similarity to infer functions but those types of data do not suffice to determine the biological context in which proteins act. Current high-throughput biological experiments produce large amounts of data on the interactions between proteins. Such data can be used to infer interaction networks and to predict the biological process that the protein is involved in. Here, we develop a probabilistic approach for protein function prediction using network data, such as protein-protein interaction measurements. We take a Bayesian approach to an existing Markov Random Field method by performing simultaneous estimation of the model parameters and prediction of protein functions. We use an adaptive Markov Chain Monte Carlo algorithm that leads to more accurate parameter estimates and consequently to improved prediction performance compared to the standard Markov Random Fields method. We tested our method using a high quality *S.cereviciae* validation network with 1622 proteins against 90 Gene Ontology terms of different levels of abstraction. Compared to three other protein function prediction methods, our approach shows very good prediction performance. Our method can be directly applied to protein-protein interaction or coexpression networks, but also can be extended to use multiple data sources. We apply our method to physical protein interaction data from *S. cerevisiae* and provide novel predictions, using 340 Gene Ontology terms, for 1170 unannotated proteins and we evaluate the predictions using the available literature.

## Introduction

Functional annotation of proteins is an important goal in post-genomics research. However, despite the many recent technological advances that have allowed the production of various types of molecular data at a genome-wide scale, the function of large numbers of proteins in fully sequenced genomes still remains unknown. This is true even for six of the most-studied model species, in which the proportion of unannotated proteins varies between 10% and 75% [1]. The general problem is that on the one hand, large-scale experimental approaches give only indirect information about the function of proteins, whereas on the other hand small-scale experiments provide more direct evidence but are labor intensive. The development of accurate computational methods for protein function prediction can therefore aid in reducing the gap between the speed of whole-genome sequencing and the functional annotation of their encoded proteomes.

**Figure 1:**
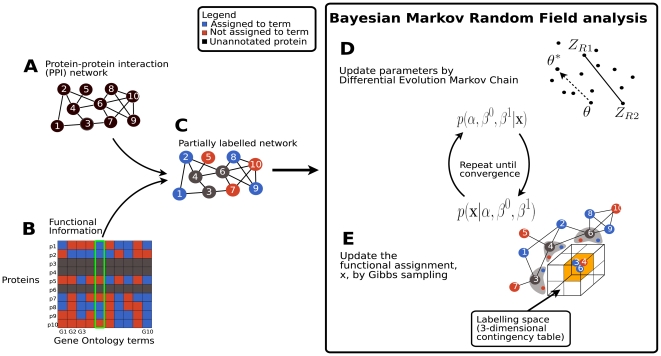
Bayesian Markov Random Fields analysis (BMRF) for protein function prediction in a nutshell. **A**. The topology of the interaction network is given. **B**. Functional annotations of proteins using a set of Gene Ontology terms. **C**. A partially annotated network. **D–E**. BMRF analysis.

The most common approach in computational prediction of protein function is to use sequence or structure similarity to transfer functional information between proteins [2]. Blast [3] and InterPro [4] searches are popular methods for such predictions. However, sequence similarity does not necessary imply functional equivalence and thus Blast based annotation transfers can be erroneous e.g. proteins from gene duplication may have high sequence similarity but different functions. Also, homology based annotation transfers lead to the percolation of misannotations in databases. Furthermore, sequence data do not provide information on the biological context of protein functions, *e.g.* the metabolic pathway or biological process that the protein is involved in. Such contextual information can be derived from large-scale data on interactions (*i.e.* physical, genetic, co-expression) between genes or gene-products, such as proteins. These data are commonly represented as networks, with nodes representing proteins and edges representing the detected interactions (Figure 1).

In a review of the existing computational methods that exploit network data for function prediction, Sharan *et. al.* [1] distinguished direct and indirect methods. Direct methods predict the function of a protein from the known functions of its neighbors (the proteins it interacts with) [5]–[9]. Indirect methods first identify functional modules in the network and subsequently assign overrepresented (enriched) functions in the module to their unannotated components [10]–[12]. Sharan *et. al.* [1] judged the direct methods as slightly superior to the indirect ones.

A pioneering direct method is the binary Markov Random Fields (MRF) method proposed by Deng *et. al.* [7] (hereafter referred to as “MRF-Deng”). In MRF-Deng, the probability that a protein performs a particular function depends on two numbers, namely the number of its direct neighbors in the network that perform the function and the number of those that do not. The parameters of this relationship are learned from a training set by logistic regression [13] using these numbers as predictors. Then, Gibbs sampling is employed for functional inference of the proteins with unknown function (“unannotated proteins”). Letovsky and Kasif (LK) [5] developed an approach that is similar to MRF-Deng, but with another parameter estimation method and with Gibbs sampling replaced by belief propagation for the prediction step. GeneMania [9] is based on a Gaussian (instead of a binary) MRF and leads to a relatively easy to solve quadratic program for making predictions.

Lanckriet *et. al.* [14] proposed an approach based on Support Vector Machines (SVM). In this approach, a similarity kernel between the proteins is computed and then a classifier is built by maximizing the margin between the proteins that perform a particular function and those that do not. The authors showed that the SVM approach leads to improved performance compared to MRF-Deng. One extension of this method is the Multi-Label Hierarchical Classification method (MLHC) [15], [16] where predictions are first made by SVM, independently per Gene Ontology (GO) [17] term, which are then made consistent with the GO hierarchy by using a Bayesian Network.

Lee *et. al.* [18] combined the appealing properties of MRF and SVM methods into Kernel Logistic Regression (KLR). Whereas the predictors in MRF-Deng are derived from the adjacency matrix that represents the network, they are derived from a similarity kernel in KLR. Parameter estimation and predictions are made by logistic regression instead of by SVM, because logistic regression is much faster. Lee *et. al.* used a diffusion kernel [19], whereby the protein neighborhoods are expanded or pruned depending on the diffusion parameter, and showed that diffusion based KLR outperforms MRF-Deng and performs comparably to diffusion kernel based SVM. In the recent experiment of [20], several state of art methods were assessed using *Mus musculus* genomic datasets leading to the conclusion that Genemania, MLHC and KLR showed appealing performance.

The application of diffusion kernel based KLR or SVM to large networks is difficult or even impossible because of the huge computational cost of the required matrix exponentiation. In this paper we therefore try to improve the original MRF-Deng method without introduction of diffusion kernels.

We discovered an important potential problem with MRF-Deng. The parameter estimation step of MRF-Deng is problematic in that proteins with known function (“annotated proteins”) have unannotated proteins as neighbors so that the predictors used in the logistic regression carry uncertainty due to the unannotated proteins (Figure 1). This problem increases with increasing numbers of unannotated proteins. MRF-Deng neglects this problem by disregarding the unannotated proteins in the first step. By this strategy, the neighborhood counts of a large number of proteins are reduced and therefore the parameter estimates tend to take larger absolute values [13]. During the Gibbs sampling, the unannotated proteins are taken into account, but the model parameters are those estimated from the pruned neighborhoods.

Here we amend the MRF-Deng method, by performing joint parameter estimation and prediction (Figure 1) as suggested by [18], [21] *i.e.* in a way that the computational cost is still modest compared to diffusion kernel based KLR. Joint analysis is a standard approach to deal with missing data in the context of semi-supervised learning and can be performed by iteratively estimating the parameters by maximizing the PseudoLikelihood Function (PLF) using logistic regression as a first step and estimating the unknown function by optimizing the objective function of the MRF in the second step, till convergence is met [22]. If there are many unannotated proteins in a given dataset then there are so many unknowns (in the second step), that optimizing them leads to a loss of statistical consistency in parameter estimation. In such cases it is much better to allow for the uncertainty therein and “average across” the unknowns [23]. We do so by taking a Bayesian approach. We model the joint posterior distribution of the model parameters and the functional states of the unannotated proteins and sample from this joint distribution by a Markov Chain Monte Carlo (MCMC) algorithm (Figure 1). We name the new method Bayesian Markov Random Field analysis (BMRF) and evaluate its performance under severe conditions, *i.e.* when half of the proteins in a network is unannotated. We show that BMRF outperforms MRF-Deng, and is competitive to diffusion KLR. Using a high quality protein-protein interaction data set of [24] we provide functional predictions for 1170 unannotated *S. cerevisiae* proteins in terms of 340 nodes (“GO terms”) of the biological process ontology of The Gene Ontology Consortium [17] and we evaluate a subset of these predictions using available literature.

## Results

### Performance Evaluation

**Figure 2:**
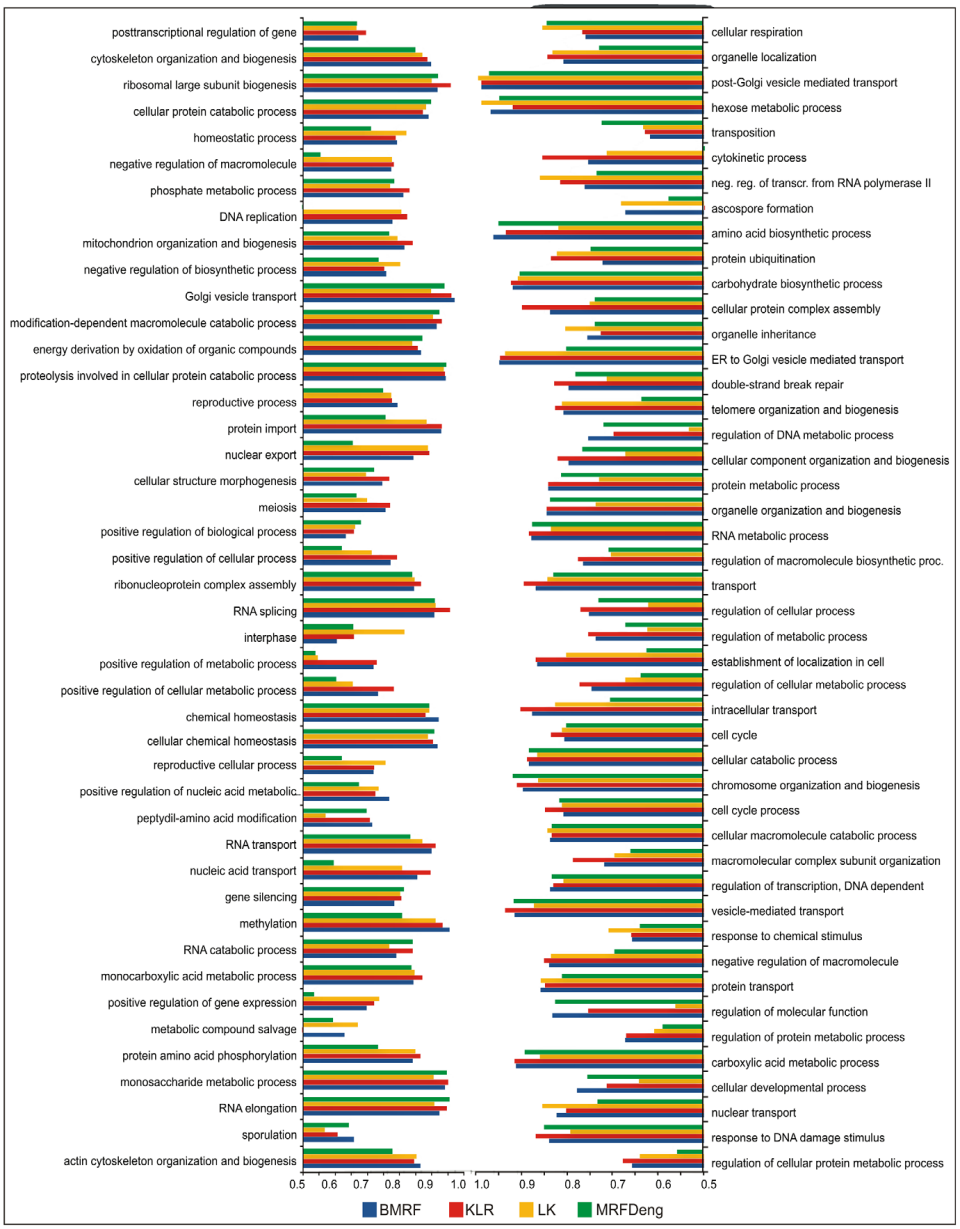
AUC scores for 90 GO terms, where the performances of the BMRF, MRF-Deng, LK and KLR was evaluated.

**Figure 3:**
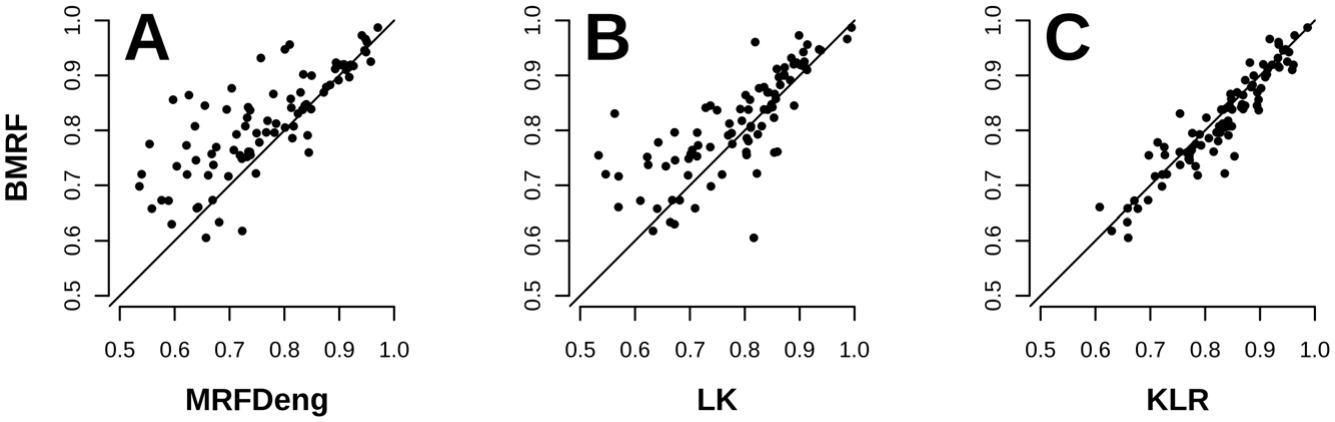
Performance comparison for 90 GO terms, using the Area Under the ROC Curve (AUC). The points above the diagonal denote improved performance of BMRF against **A**. MRF-Deng **B**. LK **C**. KLR. BMRF performs better for the majority of the tests compared to MRF-Deng and LK. KLR performs slightly better, but it is difficult to be applied in large datasets.

**Figure 4:**
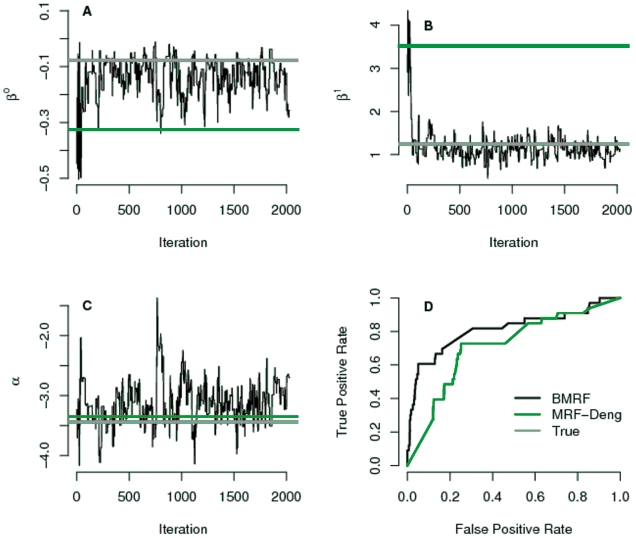
Comparison of parameter estimation and prediction performance between BMRF and MRF-Deng for the GO term “ homeostatic process”. **A–B**. In BMRF the parameters 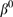 and 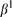 are sampled closeby to the true parameter values, in contrast to MRF-Deng where the parameters are estimated using only the annotated part of the network and lead to overestimated values. **C**. Both methods estimate the intercept reasonably well. **D**. ROC curves for the prediction performance of the two methods.The AUC value for BMRF is 0.79 and for MRF-Deng is 0.71.

We compared the prediction performance of BMRF with three other protein function prediction methods, *i.e.* MRF-Deng, LK [5] and KLR on 90 GO terms (Figure 2), by treating 800 randomly chosen proteins (out of 1622) as unannotated and using the AUC score as an indicator of the prediction performance. The AUC score denotes the probability that a randomly chosen protein that performs the function is given a higher posterior mean by the predictor than a randomly chosen protein that does not [25]. The mean AUC values for the 90 GO terms were: 0.8195 for KLR, 0.8137 for the BMRF, 0.7867 for LK and 0.7578 for MRF-Deng. BMRF performed better than LK and MRF-Deng, that served as its basis, but slightly underperformed compared to KLR (Figure 3A). The improvement of BMRF over MRF-Deng is due to the fact that BMRF estimated the interaction parameters much better. Figure 4 illustrates the parameter values based on the simulation for GO term GO:0042592 (homeostatic process). Both methods estimate the intercept parameter reasonably well (Figure 4C) but the interaction parameters (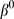 and 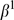) as estimated in MRF-Deng deviate far more from the true values than those of BMRF (Figure 4 AB). This led to the improvement in the prediction performance (Figure 4D). A further explanation is that the neighborhood counts of a large number of proteins are reduced in the MRF-Deng method because it disregards interactions with unannotated proteins and therefore the parameter estimates take larger absolute values. During the Gibbs sampling, the unannotated proteins are taken into account, but the model parameters are estimated from the pruned neighborhoods. This discrepancy explains the reduced performance of MRF-Deng compared to BMRF. This trend was observed for the majority of GO terms that we tested. The maximum improvement in the AUC score was 0.31 while the maximum deterioration was 0.1. We further calculated the precision when the recall is set to 20% (PR20R). The mean PR20R across all the GO terms was 0.70 for KLR, 0.62 for BMRF, 0.54 for LK and 0.31 for MRF-Deng.

**Figure 5:**
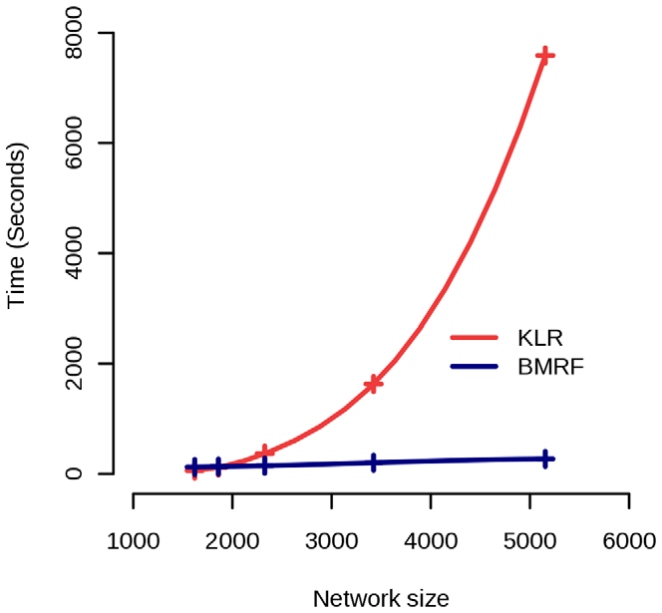
Running times for KLR and BMRF. The horizontal axis represents the size of the network and the vertical the time (in seconds) needed by each method. The computations were performed using the same hardware *i.e.* a Pentium 4 with dual core processor with 4GB of RAM and Linux operating system. The crosses denote the network size where the running times were evaluated. For BMRF the running time grows linearly with the network size while for KLR it grows polynomially.

Another important aspect of our comparison is the computational cost of the methods. BMRF has by definition larger computational cost than MRF-Deng, since it uses MRF-Deng for labelling initialization and also involves the additional parameter updating step, but the improvement in prediction performance compensates this increased cost. We did not compare with LK because our R implementation of this method was not sufficiently optimized for the speed. We compared KLR and BMRF in five networks of different sizes, constructed from the Collins *et. al.* data [24] by setting different PE score cut-offs (PE = 0.65, 1.29, 1.92, 2.55, 3.19). BMRF shows much better scaling properties and therefore is more suitable for large networks (Figure 5). The dominant factor of the computational cost of KLR is the computation of the diffusion kernel. In our implementation of KLR the diffusion kernel is obtained by scaling and squaring method with Padé approximation which is considered to be one of most competitive method currently [26]. Still, matrix exponentiation is an active field of research in Numerical Analysis and therefore faster methods or implementations may exist (i.e. the power iteration method).

### Novel Predictions for Unannotated Proteins

We applied the BMRF method for 340 GO terms, aiming to predict the functions of 1170 unannotated *S. cerevisiae* proteins. Lists of protein names, GO terms probabilities and ranks per GO term are provided as supplementary material (Table S1). We checked for further information concerning the unannotated proteins in the literature and in the Saccharomyces Genome Database (SGD, accessed during December 2008). When functional information was found, we compared it with our predictions. In the majority of cases, existing information was in accordance with our predictions (Table 1). Below we give a number of examples of these predictions and evaluations.

**Table 1:** Manually evaluated predictions of protein functions.

ORF	Protein function [reference]	Predicted GO term definition	RP Score	Rank
YNR024W	Nuclear transcribed mRNA catabolic process [27]	mRNA catabolic process	56.87	1
YDL176W	Glycolysis and gluconeogenesis [12], [28]	Glucose metabolic process	22.91	1
YMR233W	pre-mRNA 3′-end processing [29], [30]	RNA 3′-end processing	31.01	1
YOR093C	Increased levels of unfolded proteins [31]	Protein folding	28.71	1
YLR315W	Ctf19 central kinetochore complex [32]	Chromosome segregation	32.78	1
YDR383C	Ctf19 central kinetochore complex [32]	Chromosome segregation	31.68	2
YGL128C	putatively involved in pre-mRNA splicing (SGD)	Nuclear mRNA splicing, via spliceosome	43.47	1
YBL104C	nuclear mRNA splicing, via spliceosome (Blast hit)	Nuclear mRNA splicing, via spliceosome	42.68	2
YHR156C	putatively involved in pre-mRNA splicing (SGD)	Nuclear mRNA splicing, via spliceosome	41.15	3
YOR227W	endoplasmic reticulum [33] organization	Organelle organization	1.63	4
YPR003C	Transporter activity (SGD)	Ion transport	6.53	8
YKR021W	Ubiquitin-mediated endocytosis (SGD)	Cellular localization	3.65	3
YBR227C	possibly a mitochondrial chaperone (SGD)	Cation transport	8.86	1

YNR024W is involved in the degradation of “cryptic” non coding RNA [27], on the basis of which it is now annotated in SGD with a number of GO terms, including the term “nuclear-transcribed mRNA catabolic process”. In our prediction, YNR024W is indeed predicted top ranking (1st) for GO term “mRNA catabolic process” (GO:0006402) which is the parent term of the previously assigned GO term.

There is evidence that protein YDL176W is involved in glycolysis and glucoleogenesis [12], [28]. We predict this protein as top ranking (1st) in the GO term “Glucose metabolic process” (GO:0006006), which is in agreement with the existing information.

YMR233W is a Small Ubiquitin-like Modifier (SUMO) substrate [29] and in mammals is involved pre-mRNA 3′-end processing [30]. We predict the protein YMR233W to be top ranking (1st) for the GO term “RNA 3′-end processing” (GO:0031123). Targeted experiments are needed to provide more direct evidence for the role of YMR233W in mRNA processing in yeast.

YOR093C is related to increased stress levels caused by the accumulation of unfolded proteins in the endoplasmic reticulum [31]. YOR093C ranked first in “protein folding” (GO:0006457) in our predictions.

Information from SGD, based on the work of [32], reveals that YLR315W and YDR383C are non-essential subunits of the Ctf19 central kinetochore complex. The kinetochore complex is known to have a central role in chromosome segregation. In our predictions YLR315W and YDR383C ranked 1st and 2nd respectively for the term “chromosome segregation” (GO:0007059) which is in accordance with the experimental evidence.

Proteins YGL128C (1st), YBL104C (2nd), YHR156C (3rd), were co-predicted to four hierarchically dependent GO terms concerning the nuclear spliceosome mRNA splicing. They interact with proteins related to mRNA splicing in a very dense neighborhood of the protein interaction network. Information from SGD suggests that YGL156C is located in the snRNP U5 compartment and probably linked to mRNA splicing. This compartment is known to be connected with spliceosome complexes that are involved in mRNA splicing. YGL128C is annotated in SGD as putatively involved in pre-mRNA splicing, while there is an IEA annotation (Inferred from Electronic Annotation) to the RNA splicing GO term. This is a parent node of our prediction and thus we provide a more detailed prediction. Also, this protein is located in the spliceosome and therefore in principle associated with the splicing processes. SGD does not provide information on the protein YBL104C. However, using BLAST we found the protein YPR178W (e-value = 0.043) to be a distant homologue. This protein is assigned to the GO term nuclear mRNA splicing, via spliceosome and contains a splicing factor motif in its sequence. The region of similarity with YBL104C is however located outside of this motif.

YOR227W is involved in the organization of the endoplasmic reticulum [33], on the basis of which it is now annotated in SGD with the GO term endoplasmic reticulum organization. This protein ranked 4th for the GO term organelle organization (GO:0006996) which is the parent of the GO term assigned by SGD. According to SGD, YKR021W is proposed to regulate the endocytosis of the plasma membrane. This protein is top ranking for the GO term Cellular localization, which is related to the proposed function.

SGD states that YBR227C is possibly a mitochondrial chaperone with non-proteolytic function while our predictions place this protein as first ranking for cation transport. This mismatch does not necessarily imply that our prediction is false, since functional evidence from SGD can be still weak and also it is rather common that proteins have multiple functions.

## Discussion

Development of computational methods for protein function prediction based on interaction data is a challenging problem in bioinformatics. Here, we present a method to tackle this problem based on MRF. We followed the seminal work by Deng et al. (2003) in formulating the problem but we solved it in a significantly improved way. Our MCMC algorithm samples the MRF parameter values jointly with functional inference, whereas these are estimated in a single, questionable, training step in the work of [7]. Our method outperforms Dengs MRF method in efficiency of both parameter estimation and prediction performance. Also, we showed that our method performs better than the method proposed by Letovsky and Kasif [5]. The Kernel Logistic Regression (KLR) method [18] performed slightly better than BMRF, but this method involves an expensive matrix exponentiation operation, that is needed to compute the diffusion kernel. This makes KLR impractical for large networks.

In this study we focused on the methodological aspect and limit our experiments to a single data source. In this way, we could clearly show that our method is more powerful than its predecessor. Our method can handle multiple data sources such as expression correlation datasets, co-occurrence of protein names in literature obtained via text-mining, or cross-species sequence comparisons (*e.g.* orthology networks [34], [35]). The datasets can then either be merged into a single network (*e.g.* [36]), or used separately, leading to additional terms in the energy function and additional parameters ([37]) which can then be treated in the Bayesian way as proposed here. Also, protein networks for most of the species are far from complete and therefore dealing with the uncertainty of the network topology is another direction for future research.

Importantly, we showed that our approach is suitable for networks in which a large proportion of the proteins is unannotated. Our method can be applied for protein function prediction in species for which large-scale interaction datasets are available. We provided Gene Ontology predictions for 1,170 unannotated yeast proteins and for many high-ranking predictions we found supporting information in the literature.

## Methods

### Markov Random Fields

MRF methods provide the framework for probabilistic modeling of dependent random variables. They are widely applied to a variety of problems with spatial dependencies, such as image analysis [38], where a picture is considered as a square grid of pixels (*i.e* an undirected graph) and each pixel corresponds to a variable whose value (*i.e* color) depends on the values of its neighborhood pixels. In image restoration problems, MRF methods are used to restore the missing parts of the images. The most probable coloring configurations of the missing pixels can be inferred from the full joint probability distribution. The colors of the missing pixels thereby are predicted simultaneously, allowing prediction in cases where the entire neighborhoods of pixels have to be predicted. MRF is thus particularly suited for a guilt-by-association approach.

The framework for protein function prediction based on MRF was originally proposed by [7]. Given a set of N proteins and a set E of pair-wise interactions, we construct a network where nodes represent proteins and edges represent the interactions between them. Next each node is colored depending on whether the corresponding protein performs or does not perform a particular function (*e.g.* one GO term), where the coloring nodes of unannotated proteins remains unknown (Figure 1). The coloring is encoded in an N-dimensional binary vector x, *i.e.* 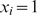 if the 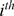 protein performs a particular function, 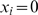, if it does not. Our aim is to assign each unannotated protein to one of the two possible states. In fact, this problem is similar to the image restoration problem described above. The MRF model entails that the probability of state 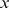 of the network given a vector 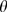 of model parameters (discussed below) is
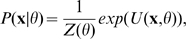
(1)where 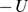 is known as the energy function and 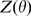 is a normalizing constant that depends on 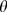. In a homogeneous second order MRF, 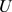 can be written as ([1], [22])
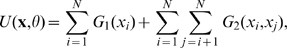
(2)where 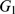 and 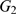 are problem-dependent functions. 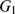 takes one value per state, without considering the interactions of the protein, *i.e.* 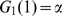 and 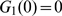. The function 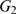 is equal to zero if proteins 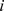 and 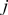 do not interact. For interacting proteins Deng *et. al.* (2003) used three classes of interactions. If both of the interacting proteins perform the function of interest then 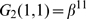. If only one of them performs the function then then 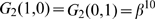, and when none of them performs the function 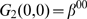. We denote the number of protein pairs in these three classes by 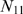, 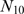 and 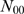, respectively. The energy function of this MRF is then 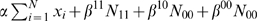, which can be rewritten in terms of the elements of 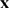 as
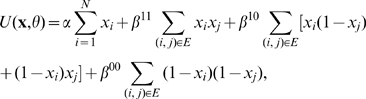
with 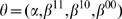. We now compare two ways of coloring the network that differ only in the value of the 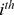 protein. By inserting equation (2) in (1) and setting 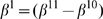 and 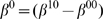, the log-odds (the logarithm of their probabilities) can be shown to be:
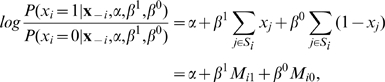
(3)where 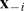 denotes 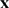 without the 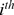 element and 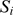 the set of proteins that interact with protein 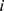. This equation is known from logistic regression. It has two predictors 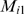 and 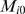 counting the number of neighboring proteins of protein 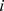 that do and do not perform the function, respectively, and three unknown parameters, whereas the function 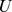 had four parameters. This is no surprise when noting that one parameter in 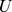 is redundant, because the sum of 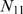, 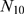 and 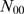 is a constant that is independent of 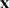. When the right-hand side of the logistic equation is a known value 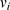, the conditional probability that unannotated protein 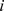 performs the function is given by the logistic function 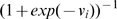. In this way we can sample the state of each unannotated protein when we know the parameters and the states of its neighbors. The problem that some or all neighbors have an unknown state can be circumvented by repeated sampling of states, starting from an initial configuration, until convergence. This process is called Gibbs sampling [38] and is performed across all unannotated proteins. Finally, the PseudoLikelihood Function (PLF) is the product of the conditional probabilities across nodes ([39])
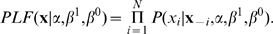



### MRF-Deng

MRF-Deng [7] consists of two tasks. In the first task, the parameters are estimated by maximizing the PLF ([39]). This can be achieved by logistic regression, in which each protein is a statistical unit, the response variable is the value of 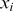 and two predictors are the numbers of neighbors of protein that do and do not perform the function. Unannotated proteins give rise to units with missing response (which are simply deleted from the regression) and to uncertain values of predictors for neighboring units (Figure 1). Thus, the two predictors cannot be precisely calculated when the neighborhood of a protein contains unannotated proteins. Consequently, the logistic regression can no longer be carried out. The authors overcame this problem by simply ignoring the unannotated proteins. In the second task, MRF-Deng makes functional inferences by Gibbs sampling across all unannotated proteins, as described above.

In summary, MRF-Deng disregards the neighborhood uncertainty in the parameter estimation step, but takes it into account during the labeling step. By disregarding unannotated proteins in the first task, neighborhoods are pruned compared to the full network. We expected that this strategy will work worse as the proportion of unannotated proteins in the network is large.

### BMRF

In this study we develop a Bayesian strategy and draw from the joint posterior density of 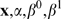 using an MCMC algorithm and starting from an initial configuration. As in [7], we will use the PLF rather than the full likelihood, as the latter has an intractable normalizing constant. A uniform prior is used as a joint prior distribution of the model parameters. The outline of our method is given in Figure 1. It is Gibbs sampling in which, at iteration, 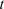, the elements of 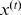 corresponding to unannotated proteins are updated conditionally on the values of the parameters 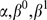, as described above, and the parameters are updated conditionally on 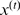. The parameter update uses the adaptive MCMC algorithm called the Differential Evolution Markov Chain (DEMC) [40] as follows. A candidate point 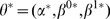 is obtained using the equation:
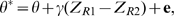
where 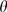 denotes the current state of the parameter vector, 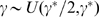 is the scaling parameter and 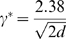 is the optimal step size [41], where 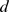 is the parameter dimension. In our problem, 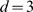 and therefore 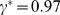. 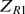, 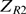 are uniformly selected from past samples of the Markov Chain as stored in a matrix 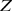 and 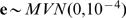. 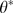 is accepted using a Metropolis step, with probability:
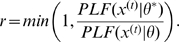



The labelling vector 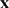 is initialized using the output of the MRF-Deng. The 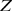 matrix is initialized in the following way. First, the Maximum Penalized Pseudolikelihood Estimates of 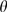, 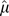 and 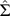 are obtained by logistic regression. We used the penalization to reduce the bias of the parameter estimates due to the small number of positive examples in the specific GO terms. Those parameter estimates were obtained using the brglm R package [42]. Then 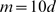 parameter values are sampled from 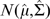 and stored in 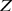, where 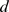 is the dimension of the parameter vector (eq 3). During the simulation, the state of 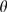 is appended to 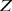 in every iteration [41]. DEMC gave near optimal acceptance rates (0.23). Convergence was tested by performing multiple independent runs from dispersed starting points. We found, by visual comparison of the posterior means of multiple runs that 2,000 iterations were sufficient to achieve convergence. The time needed for each run was around 20 seconds. The posterior probability that a protein performed the function under study was calculated by averaging the conditional probabilities that the protein performed the function, 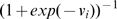, across iterations. Note that 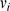 varies across iterations because parameter values and states of neighboring unannotated proteins may vary across iterations. Receiving Operating Characteristic (ROC) curves were constructed from the resulting posterior probabilities. The prediction performance was measured using the Area Under the ROC Curve (AUC) [25]. The R code of BMRF is freely available at the website: https://gforge.nbic.nl/projects/bmrf/.

### Datasets

**Figure 6:**
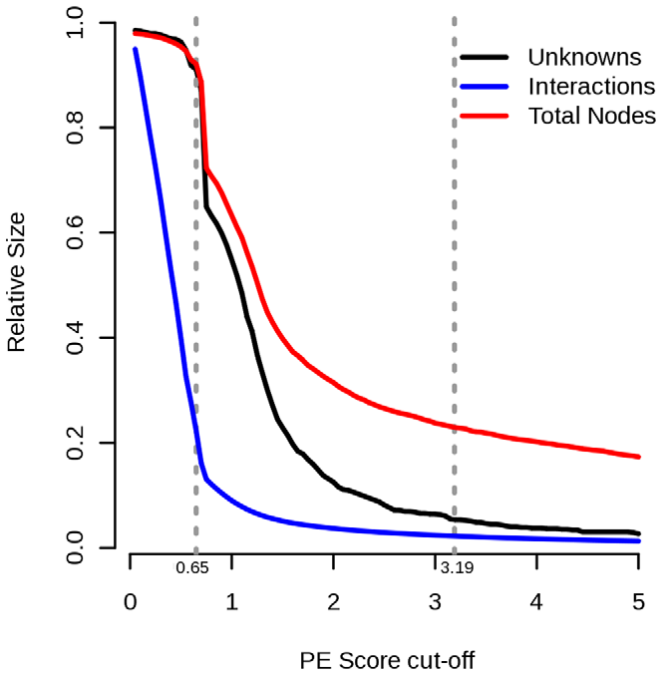
Number of unannotated proteins and number of interactions against Purification Enrichment (PE) score. The numbers are divided by their values for PE = 0 (*i.e.* the network without any cutoff that contains the full set of proteins and edges). The validation network was constructed using PE = 3.19 as suggested by [24].

We constructed a *S. cerevisiae* interaction network using the physical protein-protein interaction dataset of [24]. They used a scoring system called purification enrichment (PE) to evaluate each interaction. According to their study, selecting the interactions with PE score larger than 3.19 leads to a high quality network. This network contains 1,622 proteins (from which 84 are unannotated, corresponding to 5% of the total) and 9,074 interactions (Figure 6). We used this set of proteins and this topology as validation network for evaluating the performance of our method. Since the network provides information on the cellular process of the proteins, we used the set of GO terms that belong to the Biological Process (BP) ontology.

### Performance Evaluation

To evaluate the prediction performance of our method, we selected by stratified sampling 800 out of 1622 proteins and treated them as unannotated. This masks the annotation of about half of the proteins in the network. Such a proportion of unannotated proteins is common even for the most well studied species [1]. The originally unannotated proteins were excluded from masking, but were kept in the network. MRF-Deng and BMRF were applied to the obtained data (*i.e.* a partially labelled network, containing the masked, the unmasked proteins and unannotated proteins), resulting in posterior probabilities for each protein and for each method. The masked proteins constituted the test set and their corresponding probabilities were used to construct ROC curves and to calculate the AUC score (Figure 3). We performed “out-of-bag” evaluation on 90 GO terms (Figure 2), selected by stratified sampling across different levels of abstraction of the GO Directed Acyclic Graph. The most sparse GO term contained 21 annotated proteins, while the most general 789. We considered the parameter values as estimated from the data prior to masking as the true ones (Figure 4).

### Function Predictions for Unannotated Proteins

For actual prediction purposes we constructed an expanded network using the Collins *et. al.* [24] dataset. Figure 6, shows that for PE threshold of 0.65, most of the low confidence edges of the network are excluded while the majority of the proteins with unknown functions are included. We considered this network as suitable for protein function prediction purposes. It contained 5,419 proteins (1,170 of which were unannotated) and 89,685 interactions. The proteins assigned to the GO term biological process unknown were treated as unannotated. We applied our method to 340 GO terms from the BP ontology.

### Comparison with Other Methods

Besides MRF-Deng, we compared the performance of BMRF with two other methods for protein function prediction *i.e.* diffusion based KLR [18] and the method proposed by Letovsky and Kasif (LK) [5]. KLR performs logistic regression on the diffusion kernel of the protein interaction network.First the diffusion kernel 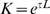 is computed, where 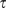 is the diffusion constant and 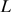 is the opposite Laplacian of the adjacency matrix of the protein interaction network. We computed 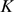 using the “expm” function of the “Matrix” R package that uses the squaring and scaling with Padé approximation. Predictions are made from the model of eq (3) using the diffusion matrix 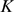 (instead of the original adjacency matrix) to define protein neighborhoods and the annotated proteins only, that is, KLR uses:
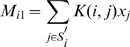


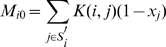
in eq (3), where 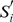 denotes the set of neighbors of protein 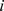 that have known function. Therefore, KLR ignores the neighborhood uncertainty in both parameter estimation and prediction, and also involves one more parameter, 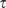. As in [18], we used a range of values for 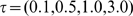 and found that the best performance was achieved for 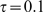 and therefore performed further computations using this value. Parameters were estimated by logistic regression. The motivation behind LK is that the number neighbors of protein 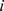 that are in state 1 is binomially distributed, conditioned on the state of the protein 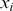. The derived model can be expressed in similar manner as eq (3). In LK inferences for the unannotated proteins of the network are made by a heuristic algorithm based on belief propagation.

### Function Predictions for Unannotated Proteins

For actual prediction purposes we constructed an expanded network using the Collins dataset ([24]). Figure 6, shows that for PE threshold of 0.65, most of the low confidence edges of the network are excluded while the majority of the proteins with unknown functions are included. We considered this network as suitable for protein function prediction purposes. It contained 5,419 proteins (1,170 of which were unannotated) and 89,685 interactions. The proteins assigned to the GO term biological process unknown were treated as unannotated. We applied our method to 340 GO terms from the BP ontology.

## Supporting Information

Table S1 Predictions of functions of unannotated proteins on a set of 346 Gene Ontology (GO) terms. The top ten ranking proteins per GO term are shown
(0.14 MB TXT)
